# Ambulatory Patients with Cardiometabolic Disease and Without Evidence of COVID-19 During the Pandemic. The CorCOVID LATAM Study

**DOI:** 10.5334/gh.932

**Published:** 2021-02-17

**Authors:** Ricardo Lopez Santi, Manlio F. Márquez, Daniel Piskorz, Clara Saldarriaga, Alberto Lorenzatti, Fernando Wyss, Alexander Valdés Martín, Jorge Sotomayor Perales, Jean Carrion Arcela, Elirub de Lourdes Rojas Gimon, Gustavo Sambadaro, Gonzalo Emanuel Perez, Ivan Mendoza, Fernando Lanas, Roberto Flores, Alvaro Sosa Liprandi, Bryce Alexander, Adrian Baranchuk

**Affiliations:** 1 Hospital Italiano de La Plata, AR; 2 Instituto Nacional de Cardiologia Ignacio Chavez, MX; 3 Cardiovascular Institute of the Rosario British Sanatorium, AR; 4 University of Antioquia, Pontificia Bolivariana University, CO; 5 Instituto Médico DAMIC/Fundación Rusculleda, AR; 6 Servicios y Tecnología Cardiovascular de Guatemala SA – Cardiosolutions, GT; 7 Instituto de Cardiología y Cirugía Cardiovascular, CU; 8 Hospital III EsSalud Juliaca, PE; 9 Hospital Almanzor Aguinaga Asenjo, PE; 10 Clinical Center, VE; 11 Sanatorio Jozama, AR; 12 Clinica Olivos, AR; 13 Tropical Cardiology, Tropical Medicine Institute, Central University of Venezuela, VE; 14 Universidad de La Frontera, CL; 15 Hospital Ramon Carrillo, AR; 16 Sanatorio Guemes, AR; 17 Queen’s University, CA

**Keywords:** Latin America, COVID-19 pandemic, cardiovascular disease, cardiometabolic disease, social determinants, SARS-CoV-2

## Abstract

**Background:**

SARS-CoV-2 pandemic has modified the cardiovascular care of ambulatory patients. The aim of this survey was to study changes in lifestyle habits, treatment adherence, and mental health status in patients with cardiometabolic disease, but no clinical evidence of COVID-19.

**Methods:**

A cross-sectional survey was conducted in ambulatory patients with cardiometabolic disease using paper/digital surveys. Variables investigated included socioeconomic status, physical activity, diet, tobacco use, alcohol intake, treatment discontinuation, and psychological symptoms.

**Results:**

A total of 4,216 patients (50.9% males, mean age 60.3 ± 15.3 years old) from 13 Spanish-speaking Latin American countries were enrolled. Among the study population, 46.4% of patients did not have contact with a healthcare provider, 31.5% reported access barriers to treatments and 17% discontinued some medication. Multivariate analysis showed that non-adherence to treatment was more prevalent in the secondary prevention group: peripheral vascular disease (OR 1.55, CI 1.08–2.24; p = 0.018), heart failure (OR 1.36, CI 1.05–1.75; p = 0.017), and coronary artery disease (OR 1.29 CI 1.04–1.60; p = 0.018). No physical activity was reported by 38% of patients. Only 15% of patients met minimum recommendations of physical activity (more than 150 minutes/week) and vegetable and fruit intake. Low/very low income (45.5%) was associated with a lower level of physical activity (p < 0.0001), less fruit and vegetables intake (p < 0.0001), more tobacco use (p < 0.001) and perception of depression (p < 0.001). Low educational level was also associated with the perception of depression (OR 1.46, CI 1.26–1.70; p < 0.01).

**Conclusions:**

Patients with cardiometabolic disease but without clinical evidence of COVID-19 showed significant medication non-adherence, especially in secondary prevention patients. Deterioration in lifestyle habits and appearance of depressive symptoms during the pandemic were frequent and related to socioeconomic status.

## Introduction

During the current COVID-19 pandemic, international organizations and national health authorities proposed a community strategy to prevent and mitigate the spread of SARS-CoV-2, and almost all countries in the world adopted social distancing measures with different degrees of restriction [1,2,3,4].

The fear of becoming infected with coronavirus, as well as the initial global public health statements urging people to stay home, led to a reduction in the number of visits and hospitalizations in patients with chronic non-communicable diseases [5,6,7]. Considering this, interest burgeoned in protocols for novel management of different pathologies [8,9,10]. This led health teams to presume that, in parallel to the COVID 19 contagion curve, there was another curve that was not being measured: one related to missed medical care in at risk populations [11].

In addition to these problems, Latin America presents a specific health issue, the impact of social determinants of health such as poverty, previously high unemployment rates, low educational level and fewer resources to combat the pandemic and its consequences [12]. These components increase the risk of social conflict and individual uncertainty with unpredictable psychological impact [13].

There is a question about the real state of affairs of patients with cardiometabolic disease but no clinical manifestation of Covid-19 in terms of lifestyle, adherence to treatments, and psychological symptoms during the pandemic [14].

## Methods

The rationale and design of the CorCOVID LATAM Study was previously published [15].

### Study population

The study population included patients with cardiometabolic disease being followed by a cardiologist in Spanish speaking Latin American countries. Eligible patients were those who did not have symptoms, signs, or suspicion of COVID-19. All gave their consent to answer a survey personally or by virtual platforms.

### Informed consent

Patients were informed about the objective of the survey and the anonymity of their responses, and gave consent to answer it personally or by virtual platforms. Ethics approval was obtained from the Interamerican Society of Cardiology (IASC) Research Ethics Board.

### Study design

A cross-sectional online survey consisting of 38 questions was developed using Google Forms (Mountain View, CA). Research staff administered the questionnaire to patients and then entered data online. In accordance with government measures to limit population mobilization, the survey was conducted either face-to-face, by phone, or by video chat, in which case informed consent was verbally taken.

The survey had seven clusters divided in two sections: 1. Questions that examine the patient’s demographic and cardiometabolic profile; 2. Questions that examine the patient’s behavior during the last 30 days. Questions contained dichotomous or multiple option answers. Answers were not forced, and respondents were permitted to select more than one response depending on the question content.

According to the World Health Organization (WHO), in its document ‘Global Recommendations on Physical Activity for Health’, physical activity is measured in minutes/week and times/week [16]. Low level of physical activity was considered as 2 times or less/week and less than 100 minutes/week, too.

Regarding fruits and vegetables consumption we measured in days/week and servings/day as per WHO. The international goals are 400 gr/day distributed in 4–5 servings/day (2 of fruit and 3 of vegetables). We considered very low intake less than three days/week and less than three servings/day.

### Study distribution

The Interamerican Society of Cardiology opened the call for cardiologists of Spanish speaking Latin American countries to join as researchers on June 1st, 2020. Sixty-six investigators from 13 countries (divided in three geographic regions) applied and were approved:

Region 1 (North, Central, and Caribbean region): Costa Rica, Cuba, El Salvador, Guatemala, México, and República Dominicana.

Region 2 (Andean region): Colombia, Ecuador, Perú, and Venezuela.

Region 3 (Southern cone region): Argentina, Chile, and Paraguay.

The survey platform was open between June 15 and July 15, 2020 for the investigator team. Reminders were emailed daily to maximize the response rate, with information about the progression of the total surveys by country, as an incentive.

### Statistical analysis

Data was described using means and standard deviations for continuous variables, and frequencies and percentages for categorical variables. Independent sample *t*-tests were used to compare the normally distributed continuous variables, the Mann-Whitney *U* test was used for non-normally distributed continuous variables, and the Pearson’s chi-squared test (or the Fisher’s exact test as appropriate) for categorical variables. A *P* value of less than 0.05 was considered statistically significant. Multiple logistic regression models were constructed for comparisons, OR and 95% confidence intervals (CI) were provided to establish which variables are determining factors in the main negative changes observed in this population during the SARS-COV 2 outbreak. Data was collected in Google Forms. All statistical analysis was performed using Stata v. 13.1 (Stata Corp LP, USA).

## Results

**Figure 1:**
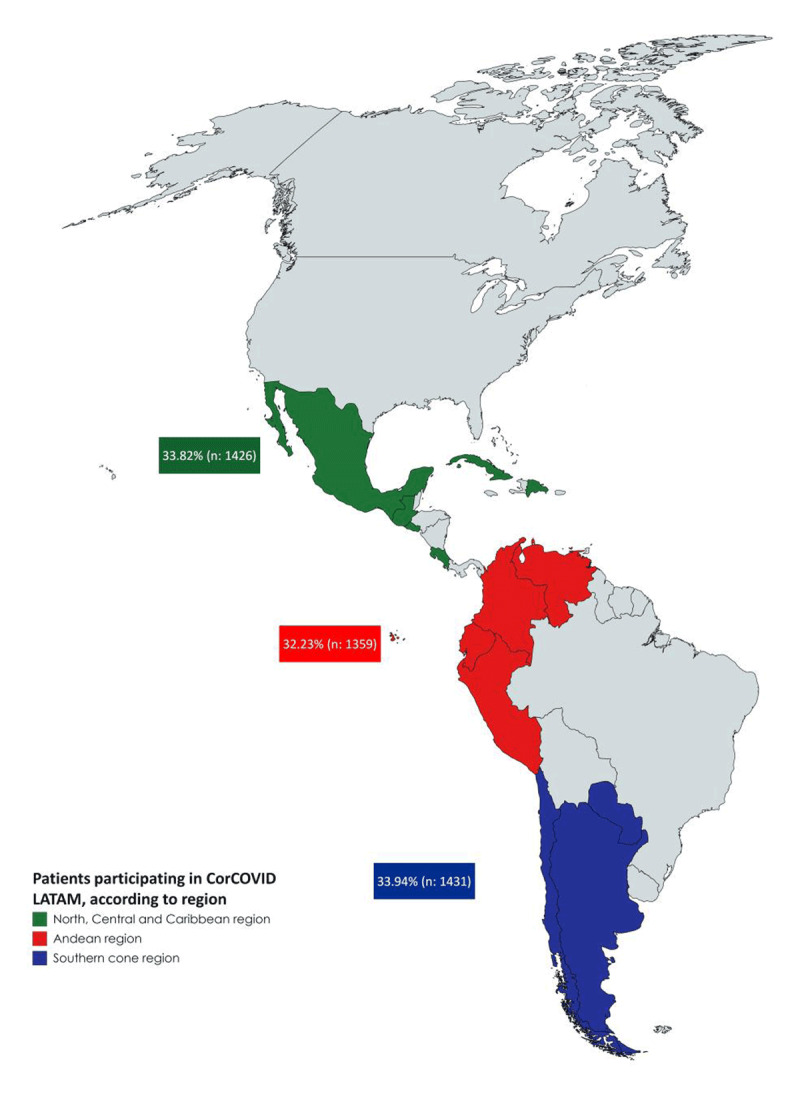
Distribution of surveyed by region.

A total of 4429 surveys(s) were collected, 213 of which were discarded due to incomplete data, duplicate document, or non-meeting inclusion criteria. As such, 4216 were included in the database for statistical analysis distributed by regions as shown in Figure 1. The mean age of the population was 60.35 ±15.39 years old, and 2147 patients were men (50.9%).

Baseline socio-demographic characteristics are listed in Table 1.

**Table 1:** Descriptive analysis of socio-demographic characteristic of the population. Categorical variables are shown as percentages with counts in parentheses.

Baseline socio demographic profile		Baseline habits profile	

***Region of origin***		***Habits***	
➢ North, Central, Caribbean	33.8% (1426)	**Smoking**	
➢ Andean	32.2% (1359)	➢ No-smoking	85.6% (3610)
➢ Southern cone	33.9% (1431)	➢ 1–10 cigarettes/day	9.0% (381)
***Age (years)***	60.3 (SD 15.39)	➢ 10–20 cigarettes/day	3.9% (165)
***Sex***		➢ More than 20 cigarettes/day	1.4% (60)
➢ Male	50.9% (2147)	**Physical activity**	
➢ Female	49% (2069)	➢ None	38% (1605)
***Education***		➢ Less than 3 times/week	34.4% (1454)
➢ None	2.2% (96)	➢ 3–6 times/week	14.9% (632)
➢ Primary	19.1% (807)	➢ Everyday	12.4% (525)
➢ Secondary	32.1% (1355)	**Physical activity Min/week**	
➢ Tertiary	15.3% (646)	➢ Less than 100 minutes	44.5% (1164)
➢ Academic	31.1% (1312)	➢ 100 to 150 minutes	31.3% (818)
***Occupation***		➢ 151 to 200 minutes	14.2% (372)
➢ Housekeeper	4.6% (194)	➢ More than 200 minutes	9.8% (257)
➢ Public employee	18.1% (766)	**Alcohol intake**	
➢ Private employee	19.4% (819)	➢ Yes	42.6% (1797)
➢ Independent contractor	6.7% (284)	➢ No	57.3% (2419)
➢ Academic degree	12.1% (512)	**Frequency of consumption (n = 1797)**	
➢ Retired	12.5% (528)	➢ Occasionally	68.2% (1227)
➢ Unemployed	26.4% (1113)	➢ Every week	17.2% (310)
***Income level***		➢ Almost every day	7.7% (139)
➢ Very low	10% (422)	➢ Every day	6% (109)
➢ Low	35.5% (1498)	➢ Missing data	0.6% (12)
➢ Middle	48.5% (2047)	**Frequency of intake of fruits and vegetables**	
➢ High	5.9% (249)	➢ Never	7.7% (326)
		➢ 1–3 days/week	34.8% (1469)
		➢ 4–6 days/week	22.7% (958)
		➢ Everyday	34.7% (1463)
		**Daily intake of fruits and vegetables**	
		➢ 1 serving	28.5% (1202)
		➢ 2–3 servings	56.7% (2393)
		➢ 4–5 servings	10.7% (451)
		➢ More than 5 servings	2.6% (111)
		➢ Missing data	1.4% (59)

### Socio-economic and cardiometabolic profile

Among this population, 1920 (45.54%) belonged to the low and very low incomes group and 903 (22.4%) had primary education level or non-education.

Table 2 shows the cardiometabolic profile of the study population.

**Table 2:** Descriptive analysis of cardiometabolic profile of the population. Categorical variables are shown as percentages with counts in parentheses.

Baseline Cardiometabolic profile	

***CV History***	
Hypertension	72.8 % (3071)
Dyslipidemia	36.8 % (1555)
Diabetes	21.3 % (899)
Coronary disease	18.2 % (769)
Arrhythmias	16.9 % (714)
Heart failure	10.3 % (436)
Valvulopathy	8.2 % (346)
Peripheral vascular disease	4.2 % (181)
Cardiomyopathy	4 % (172)
Stroke	3.9 % (168)
Cardiac devices	3.8 % (164)
Other	2.3 % (99)
***Last hospitalization due to a cardiovascular event***	
Never	51.3% (2165)
2020	11.1% (470)
2019	11.1% (469)
2018	7.0% (298)
2017 or earlier	19.3% (814)
***Number of pills taken per day***	
Less than 4	67.0% (2825)
5 to 8	27.5% (1160)
9 to 12	4.8% (203)
More than 12	0.6% (28)

Among risk factors, hypertension (3071/72.8%), dyslipidemia (1555/36.8%) and diabetes (899/21.3%) were the most frequent.

Coronary artery disease (CAD) (769/18.2%) and heart failure (436/10.3%) were the most prevalent secondary prevention population.

Of the population, 1391 (32.9%) took 5 or more pills daily at the time of the survey and 2258 (53.56%) had at least one contact with the health team in the last month.

### Lifestyles habits

Among the total population, 2611 (62%) performed physical activity, but only 629 (14.9%) met the minimum recommendations of 150 minutes per week. Only 562 (14.7%) had the recommended daily intake of four or more servings. Finally, 1797 (42.6%) were reported as habitual drinkers and 606 (14.4%) as current smokers.

### Changes during the pandemic

The major changes in self-reported lifestyle habits are shown in Table 3, classified into 4 main items: physical activity, smoking, alcohol intake, and dietary intake of fruits and vegetables.

➢ Physical activity and diet

**Table 3:** Changes in lifestyle habits in the last 30 days. Behavior is expressed as percentage of affirmative answers and in brackets the number of patients involved.

Physical activity	n (%)

Changes in physical activity (n = 2611)	
It has been less than in the previous months	61.3% (1601)
It has been the same as in the previous months	28.4% (742)
It has been more than in the previous months	10.2% (268)
Have you used any app for training?	
Yes	21.3% (558)
No	78.6% (2053)
**Smoking**	

Changes in Smoking habit (n = 606)	
It has been less than in the previous months	33% (200)
It has been the same as in the previous months	25.7% (156)
It has been more than in the previous months	17% (103)
Missing data	24.2% (147)
Have you considered Smoking cessation?	
Yes	52.3% (317)
**Alcohol Intake**	

Changes in alcohol intake (n = 1797)	
It has been less than in the previous months	45.4% (816)
It has been the same as in the previous months	34.7% (624)
It has been more than in the previous months	11.3% (204)
Missing data	8.5% (153)
**Dietary intake of fruits and vegetables**	

Changes in fruits and vegetables intake (n = 4216)	
It has been less than in the previous months	27.9% (1177)
It has been the same as in the previous months	49.8% (2103)
It has been more than in the previous months	22.2% (936)

Among the population who regularly perform physical activity, 1601 (61.3%) reported less volume than the previous months.

**Figure 2:**
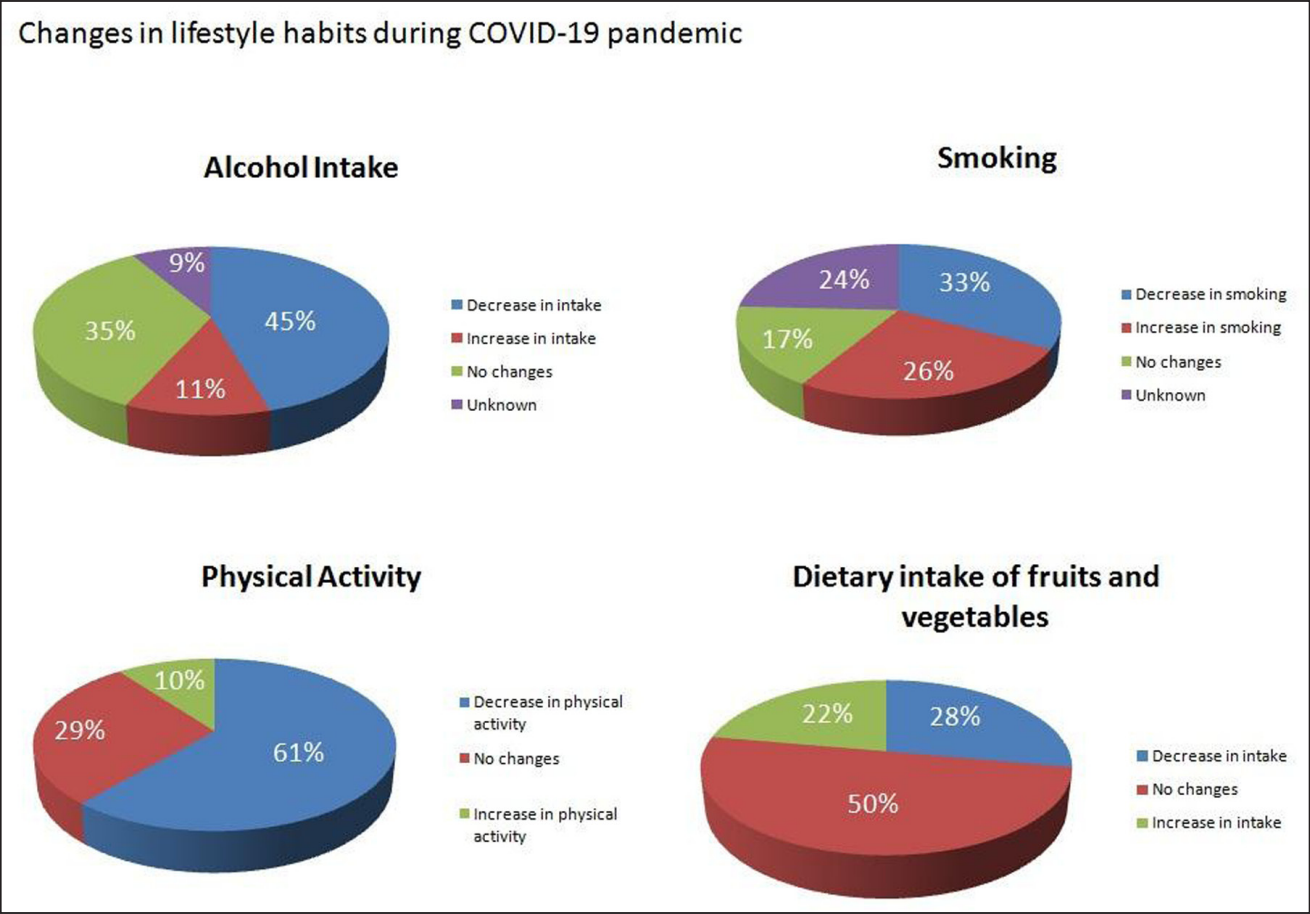
Main changes in lifestyle habits.

The surveyed population reported a low consumption of fruits and vegetables 1795 (42.5%) 3 or fewer days/week and 3595 (85.5%) 3 or fewer servings/day. In the preceding 30 days, vegetable and fruit intake diminished in 27.9% of the surveyed population, did not change in 49.8% and increased in 22.2% of the patients, (Figure 2).

➢ Tobacco and Alcohol

In the current smoking population (103), 17% stated an increase in tobacco consumption but, as a positive finding, 52.3% reported they had considered quitting tobacco.

In the regular drinker population (1797), 11.3% stated they drank more, while 79% stated they drank the same or less quantity than they did before the pandemic.

### Psychological symptoms

The perception of depression was reported by 1751 (41.5%) and was associated with low educational level (OR 1.46 CI 1.26–1.70; *P* < 0.01).

Other psychological symptoms were reported: weariness (2219/52.6%), decreased attention (1219/28.9%), sleeplessness or insomnia problems (2049/48.6%), and loss of interest in usual activities (3349/79.4%).

Univariate analysis showed an association between the perception of depression and low income (OR 0.48 CI 0.42–0.55; *P* < 0.001), sedentary lifestyle (OR 0.62 CI 0.54–0.70; *P* < 0.001), decreased food intake (OR 0.48 CI 0.43–0.57; *P* < 0.001) and perception of weight loss (OR 0.48 CI 0.41–0.56; *P* < 0.001).

Low-income was associated with a lower level of physical activity (*P* < 0.0001), less consumption of fruits and vegetables (*P* < 0.0001), more tobacco use (*P* < 0.001), and a higher perception of depression (*P* < 0.001).

### Adherence to pharmacological treatments

**Figure 3:**
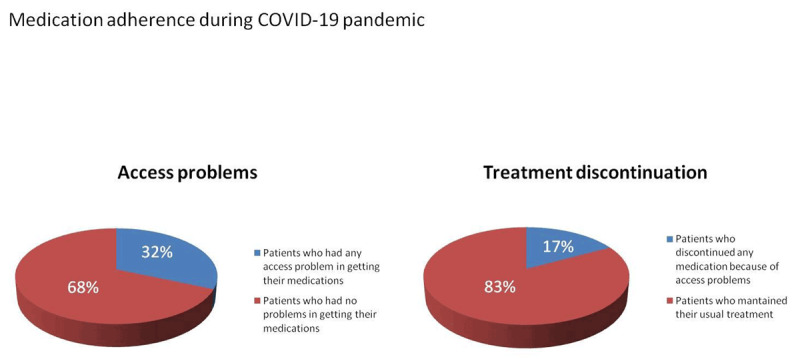
Percentage of patients who have had problems accessing some medication and patients with treatment discontinuation.

A total of 1330 patients (31.5%) reported access barriers to pharmacological treatments, and 720 (17%) reported discontinuation of some medication (Figure 3). After applying multivariate analysis, secondary prevention status was significantly associated with this situation: peripheral disease (OR 1.55 CI 1.08–2.24; *P* = 0.018), heart failure (OR 1.36 CI 1.05–1.75; *P* = 0.017), valvular heart disease (OR 1.66 CI 1.27–2.17; *P* < 0.001) and CAD (OR 1.29 CI 1.04–1.60; *P* = 0.018).

Perception of depression increased chances of discontinuing a medication (OR 2.01 CI 1.71–2.37; *P* < 0.001).

Details of univariate and multivariate analysis are listed in Table 4.

**Table 4:** Discontinuation of medication related to numbers of pills and cardiovascular antecedents.

	Univariate Logistic			Multivariate Logistic		

	OR crude	95%CI	*P* value	Adjusted OR	95%CI	*P* value

Male sex	0.92	0.78–1.08	0.312	1.01	0.86–1.20	0.819
Pills per day						
- Less than 4	Baseline	N/A	N/A	Baseline	N/A	N/A
- 5–8	0.85	0.70–1.02	0.096	0.80	0.65–0.99	0.047
- 9–12	1.19	0.83–1.70	0.335	1.07	0.73–1.59	0.698
- More than 12	1.28	0.51–3.18	0.590	1.09	0.42–2.80	0.857
**Cardiovascular History**						

Stroke	0.72	0.45–1.14	0.163	0.70	0.44–1.12	0.147
**Peripheral vascular disease**	**1.54**	**1.08–2.19**	**0.015**	**1.55**	**1.08–2.24**	**0.018**
**Coronary disease**	**1.16**	**0.94–1.41**	**0.148**	**1.29**	**1.04–1.60**	**0.018**
Diabetes	1.01	0.83–1.23	0.883	1.08	0.88–1.33	0.411
Hypertension	0.74	0.62–0.88	0.001	0.86	0.71–1.03	0.119
Dyslipidemia	0.81	0.68–0.96	0.016	0.85	0.71–1.02	0.084
**Heart failure**	**1.44**	**1.13–1.84**	**0.003**	**1.36**	**1.05–1.75**	**0.017**
Cardiomyopathy	1.49	1.04–2.15	0.029	1.30	0.89–1.91	0.172
**Valvular heart disease**	**1.77**	**1.37–2.29**	**<0.001**	**1.66**	**1.27–2.17**	**<0.001**
Arrhythmias	0.97	0.78–1.21	0.833	0.90	0.72–1.12	0.357
Cardiac devices	1.04	0.69–1.57	0.834	0.95	0.62–1.45	0.816
**Depression**	**2.02**	**1.72–2.38**	**0.000**	**2.01**	**1.71–2.37**	**<0.001**

## Discussion

The main finding of our work was to demonstrate that a significant number of patients with cardiometabolic disease showed worsening of lifestyle habits, inadequate medical follow-up, frequent discontinuation of medications, and a worrying incidence of psychological symptoms. This was particularly apparent in the secondary prevention group.

A recent editorial by Robert Brook et al. analyzed the impact of major catastrophes on cardiometabolic risk factors. The authors proposed a strategy of anticipating, not just reacting to, the possible coming of a second crisis derived from the clinical worsening of cardiometabolic patients and suggested possible tools to deal with the problem [17]. The contribution of the Cor-COVID-LATAM survey aims to further these goals.

It behooves us to consider that inadequate follow-up increases the possibility of not detecting asymptomatic or minimally symptomatic disturbances in high-risk patients. Among our surveyed cardiometabolic population, 1958 (46.4%) did not have any type of contact with a healthcare provider despite the widely spread recommendations of not neglecting the care of chronic pathology. This situation is consistent with what has already been reported in Asia and Europe [18,19].

Despite the current availability of virtual tools to guide patients in diet and physical activity, the health system was not prepared to respond adequately in a short time to reach thousands of patients [20]. Thus, only 558 (21.3%) among those who performed physical activity used web tutorials or apps. Inexperience in the use of telemedicine tools, and in many cases the non-existence of updated patient databases, increased the lack of connection between the health teams and patients with cardiometabolic disease, particularly in public health systems belonging to low and middle incomes countries [21,22,23]. The absence of adequate follow-up of patients lead to missed opportunities, such as that offered by 52.3% of active smokers, who reported that they had contemplated quitting smoking [24].

A relevant result of this study is the important number of patients discontinuing treatment. As it is well known, this increases the risk of events in a short period of time [25,26]. During the last decade, adherence to cardiovascular treatment guidelines has been a highly debated topic [27]. Some evidence has shown that patients’ self-reports usually underestimate adherence issues. The FOCUS study demonstrated that adherence falls from 68% to 50.8% when investigators add pill count to the Morisky Green Test, a short survey that evaluates adherence [28]. Based on this, it is likely that non-adherence could be greater than that reported by patients in this study.

The maintenance of cardiovascular treatment is a complex challenge with different components. Among these, access barriers emerged as a major finding as 1330 (31.5%) reported difficulties in obtaining some medication. Similarly, data from the Prospective Urban and Rural Epidemiological (PURE) Study showed that in Latin American countries, secondary prevention drugs may not be available or affordable for a large proportion of communities and households [29]. For instance, at the time of this survey, most Latin American countries did not accept digital prescription of drugs, giving rise to a new barrier to access medication given the mobility restriction measures.

Another important aspect affecting cardiometabolic patients’ treatments was the controversial information in the press and social media about some medicines (e.g.: angiotensin-converting enzyme inhibitor or angiotensin receptor blockers) as a potential gateway for the coronavirus [30,31]. Despite the immediate position statement of many cardiovascular scientific societies, including the Inter American Society of Cardiology, strongly recommending to continue cardiovascular treatments, it did not completely avoid a lack of adherence to these treatments [32].

Since the beginning of the outbreak of SARS-CoV-2, potential psychological impact was of great concern [33,34,35]. This study demonstrated a significant percentage of patients reporting psychological symptoms. Among them, the perception of depression showed an association with social determinants of health such as low-income and educational level, factors that have previously been shown to enhance cardiovascular mortality [36]. We recently published the most detailed results regarding psychological impact and 1,590 individuals (37.71%; IC95% 36.24–39.19) were considered suffering major depression independently associated with female gender (OR 1.72; 95%CI 1.40–2.11; p < 0.0001), low physical activity <100 minutes weekly (OR 1.36; 95%CI 1.10–1.67; p < 0.004), and low fruits and vegetables intake (OR 1.46; 95%CI 1.05–2.03; p < 0.024) [37]. The consequences of these neglected aspects will surely be reflected in an increase in mental disorders such as major depression and stress, which have a proven relationship with cardiovascular outcomes [38]. A recent web survey of the UK Household Longitudinal Study (UKHLS) showed that mental health deteriorated compared to pre-COVID-19 times, and the authors commented on the need to generate policies that address special populations such as women, youth, and children in order to prevent future mental illnesses [39]. Early detection and proper treatment of depression are crucial aspects to start the resilience process [40].

## Limitations

Among the limitations of this study are the absence of a random sample design to achieve a better representativeness of the participating countries’ population. Even though we were able to include more than 4000 patients, this is only a partial view of the continental situation, and probably, there is high regional and local heterogeneity. However, a significant number of surveys in a short period of time has allowed us to create a snapshot that reasonably expresses the situation of cardiometabolic patients in Latin America. Another limitation is that Brazil was not included in the survey, as the survey was limited to Spanish speaking countries. Finally, the self-reported variables may lead to bias, related to the type of survey and psychological status of patients during the interview.

## Conclusions

A significant proportion of the surveyed Latin American patients with cardiometabolic disease but without clinical evidence of SARS-CoV-2 infection during the ongoing COVID-19 pandemic showed a worsening of lifestyle habits, inadequate medical follow-up, frequent discontinuation of medications, particularly in the secondary prevention group, and worrying incidence of psychological symptoms. Some of them were related to low income and educational levels. These findings show the greater vulnerability of individuals living in middle-income countries during this unprecedented pandemic.

## Additional Files

The additional files for this article can be found as follows:

10.5334/gh.932.s1 Appendix 1. CorCOVID Survey.

10.5334/gh.932.s2 Appendix 2. Investigators.
