# Supplementary material for: Ambulatory Patients with Cardiometabolic Disease and Without Evidence of COVID-19 During the Pandemic. The CorCOVID LATAM Study
Source: Glob Heart. 2021 Feb 17;16(1):15. doi: 10.5334/gh.932 (PMC7894376; doi:10.5334/gh.932)
Supplement: Appendix 1. — CorCOVID Survey. [file gh-16-1-932-s1.pdf]

SIAC
Sociedad Interamericana de Cardiología

SIAComunidad

Status of patients with cardiovascular disease in the pandemic context

CorCOVID LATAM

Initials:

Date of birth:

Sex:

- *Male*
- *Female*

Education level

- *None*
- *Primary school*
- *High school*
- *Tertiary*
- *College*

Occupation

- *Housekeeper*
- *Employee*
- *Independent contractor*
- *Academic degree*
- *Retired*
- *Unemployed*

Income level

- *Very Low (NOT COVER BASIC NEEDS AND REQUIRES SOCIAL SUPPORT)*
- *Low (COVER ONLY BASIC NEEDS AND MEDICATIONS)*
- *Middle (EXCEEDS BASIC NEEDS AND ALLOWS SAVINGS)*
- *High*

Size of your home based on number of rooms

- *1*
- *2*
- *3*
- *4*
- *5 or more*

How many people do you live with?

- *None*
- *1 ó 2*
- *3 ó 4*
- *5 ó 6*
- *7 or more*

Cardiovascular history

- *Stroke*
- *Peripheral vascular disease*
- *Coronary disease*
- *Diabetes*
- *Hypertension*
- *Dyslipidemia*
- *Heart failure*
- *Cardiomyopathy*
- *Valvular heart disease*
- *Arrhythmias*
- *Cardiac devices*
- *Other*

Last hospitalization due to a cardiovascular event

- *Never*
- *2020*
- *2019*
- *2018*
- *2017 or Earlier*

Number of pills taken per day

- *1 to 4*
- *5 to 8*
- *9 to 12*
- *More than 12*

THE FOLLOWING QUESTION REFER TO THE LAST 30 DAYS

How often have you been physically active? (Any activity that involves movement including dance)

- *Never*
- *Less than 3 times per week*
- *3 or more times per week*
- *Everyday*

Weekly amount in minutes of physical activity

- *Less than 100 minutes*
- *100 to 150 minutes*
- *150 to 200 minutes*
- *More than 200 minutes*

Physical activity made

- *It has been less than in the previous months*
- *It has been the same as in the previous months*
- *It has been more than in the previous months*

Have you used any tutorial or phone app for training?

- *Yes*
- *No*

How often have you smoke?

- *None*
- *1 to 10*
- *10 to 20*
- *More than 20*

If you have smoked

- *It has been less than in previous months*
- *It has been the same as in previous months*
- *It has been more than in previous months*

If you have smoked. Have you considered Smoking cessation because of Coronavirus?

- *Yes*
- *No*

Did you consume any kind of alcoholic drink?

- *Yes*
- *No*

What kind of alcoholic drink?

- *Wine*
- *Beer*
- *Spirit*
- *Other*

How often did you drink alcoholic beverages?

- *Occasionally*
- *Every week*
- *Almost everyday*
- *Everyday*

If you have drunk

- *It has been less than in previous months*
- *It has been the same as in previous months*
- *It has been more than in previous months*

Have you eaten fruits and vegetables?

- *Rarely*
- *1 to 3 times per week*
- *4 to 6 times per week*
- *Everyday*

How many servings of fruits and vegetables have you eaten in one day (average)?

- *1*
- *2 or 3*
- *4 or 5*
- *More than 5*

How much food have you eaten (all types of food)?

- *It has been less than in previous months*
- *It has been the same as in previous months*
- *It has been more than in previous months*

Are you weighing yourself at home?

- *Yes*
- *No*

In your perception

- *Has gained weight*
- *My weight has not changed*
- *Has lost weight*

Have you had any problems accessing your medication?

- *Yes*
- *No*

Have you stopped any medications due to problems getting them?

- *Yes*
- *No*

Have you received flu shot?

- *Yes*
- *No*

Have you received pneumococcal vaccination in the last 3 years?

- *Yes*
- *No*

Have you made any medical consultation in person, by phone or computer?

- *Yes*
- *No*

Have you done new activities this month like arts and crafts, reading books, hobbies?

- *Yes*
- *No*

Have you been interested in activities you usually like to do?

- *Yes*
- *No*

Have you been feeling depressed?

- *Yes*
- *No*

Have you felt tired or less energetic?

- *Yes*
- *No*

Have you had trouble concentrating on work or watching television?

- *Yes*
- *No*

Do you look for pandemic news on TV or online newspapers?

- *Never*
- *1 to 3 times per week*
- *4 a 6 times per week*
- *Everyday*
- *Several times each day*

Have you had trouble getting a proper rest due to wakefulness, insomnia?

- *Yes*
- *No*

When you wake up in the morning. Do you feel that you rested well?

- *Yes*
- *No*
